# Iron-chelating agent desferrioxamine stimulates formation of neutrophil extracellular traps (NETs) in human blood-derived neutrophils

**DOI:** 10.1042/BSR20160031

**Published:** 2016-05-20

**Authors:** Lena Völlger, Kathryn Akong-Moore, Linda Cox, Oliver Goldmann, Yanming Wang, Simon T. Schäfer, Hassan Y. Naim, Victor Nizet, Maren von Köckritz-Blickwede

**Affiliations:** * Department of Physiological Chemistry, University of Veterinary Medicine Hannover, 30559 Hannover, Germany; † Department of Pediatrics, UCSD School of Medicine, San Diego, La Jolla, CA 9500, U.S.A.; ‡ Klinik für Anästhesiologie und Intensivmedizin, Universitätsklinikum Essen, Universität Duisburg-Essen, 45147 Essen, Germany; § Institut für Physiologie, Universität Duisburg-Essen, 45147 Essen, Germany; ║ Infection Immunology Group, Helmholtz Centre for Infection Research, 38124 Braunschweig, Germany; ¶ Center for Eukaryotic Gene Regulation, Department of Biochemistry and Molecular Biology, Pennsylvania State University, University Park, PA 16802, U.S.A.; ** Skaggs School of Pharmacy & Pharmaceutical Sciences, University of California, San Diego, La Jolla, CA 92093, U.S.A.; †† Research Center for Emerging Infections and Zoonoses (RIZ), University of Veterinary Medicine Hannover, 30559 Hannover, Germany

**Keywords:** extracellular traps, innate immunity, neutrophils

## Abstract

Neutrophil extracellular trap (NET) formation is a significant innate immune defense mechanism against microbial infection that complements other neutrophil functions including phagocytosis and degranulation of antimicrobial peptides. NETs are decondensed chromatin structures in which antimicrobial components (histones, antimicrobial peptides and proteases) are deployed and mediate immobilization of microbes. Here we describe an effect of iron chelation on the phenotype of NET formation. Iron-chelating agent desferrioxamine (DFO) showed a modest but significant induction of NETs by freshly isolated human neutrophils as visualized and quantified by immunocytochemistry against histone–DNA complexes. Further analyses revealed that NET induction by iron chelation required NADPH-dependent production of reactive oxygen species (ROS) as well as protease and peptidyl-arginine-deiminase 4 (PAD4) activities, three key mechanistic pathways previously linked to NET formation. Our results demonstrate that iron chelation by DFO contributes to the formation of NETs and suggest a target for pharmacological manipulation of NET activity.

## Abstract (precis)

Here we show that iron-chelating agent desferrioxamine significantly induced the formation of neutrophil extracellular traps by human blood-derived neutrophils as visualized and quantified by immunofluorescence microscopy. Further analyses characterized biochemical mechanisms associated with the NET formation by desferrioxamine.

## INTRODUCTION

Neutrophils are part of the first line of defense against pathogens within the innate immune system. These specialized leucocytes support a variety of intra- and extracellular antimicrobial functions and collaborate in processes of tissue remodelling and tissue repair. Three principal modes of neutrophil antimicrobial function are known: phagocytosis, degranulation and extracellular trap (ET) formation [1]. ETs are released by different immune cells such as neutrophils [2], macrophages/monocytes [3], mast cells [4] and eosinophils [5] after stimulation with host cytokines or microbial-derived factors. Neutrophil ETs (NETs) consist of a backbone of DNA fibres, closely associated with antimicrobial peptides, histones and proteases which exert antimicrobial activity [2,6]. These structures have been shown to entrap and immobilize several bacterial and fungal pathogens [1,2,7–9] and thereby restrict their spread within the infected host.

The cellular processes that lead to the formation of NETs are not fully elucidated. Different inflammatory mediators like interleukin-8 (IL-8) [2], interferon (IFN) α/γ [10], phorbol myristate acetate (PMA) [2] or hydrogen peroxide (H_2_O_2_) [6] are strong NET stimulants, and direct exposure to bacteria or fungi [6,11–14] or bacterial-derived factors such as lipopolysaccharide (LPS) [2] can induce NET formation. These signals can lead to NADPH-oxidase activation and formation of reactive oxygen species (ROS), which has been shown to be essential for NET formation in response to some stimuli e.g. bacteria [6], and contribute to NET formation with others, e.g. enrofloxacin [15]. Ultimately, the nuclear membrane of the activated neutrophil dissolves and chromatin is decondensed. The disrupted nuclear membrane allows nuclear components to mix with the cytoplasmic granular proteins of the cell [6]. Finally those mixed nuclear and cytoplasmic components are released into the extracellular space as ETs. In 2009 Wang et al. [16] showed that histone hypercitrullination by peptidyl-arginine-deiminase 4 (PAD4) regulates the unfolding of chromatin during the formation of NETs. Furthermore, neutrophil elastase has been shown to proteolytically modify histones and contribute to NET formation [17].

Dysregulation of NET formation is thought to have serious consequences: as reviewed by Saffarzadeh and Preissner [18], excessive NET formation can, e.g. trigger autoimmune diseases or thrombosis, and insufficient NET formation can lead to an ineffective defense against infection. Understanding the mechanisms that regulate NET formation might lead to potential drug targets for treating infections or autoimmune diseases. Since treatment of neutrophils with iron-chelating agents has been described to alter their antimicrobial activities [19], we aimed to study the effect of iron-chelation on formation of NETs. Here we show that iron-chelating agent desferrioxamine (DFO) leads to the formation of NETs in human blood-derived neutrophils.

## MATERIALS AND METHODS

### Bacterial strains

*Staphylococcus aureus* strain LAC (pulsed-field type USA300), a community-acquired CA-MRSA strain [20], was used for entrapment studies as previously described [21].

### Microscopy to visualize the effect of DFO and L-mimosine on NET formation

Human neutrophils were isolated from fresh heparinized blood by density centrifugation at 500 x ***g*** using Polymorphprep™ (Axis-Shield PoC). Neutrophils have been seeded on cover slides covered with 0.01% Poly-L-lysine (# P4707, Sigma–Aldrich).

A total of 5×10^5^ cells in 250 μl RPMI 1640 (# E15-848, phenol red free, PAA) were seeded per well in a 24-well-plate. The cells were either stimulated with 25 nM PMA (Sigma–Aldrich), or 300 μM DFO (D9533, Sigma–Aldrich) for 3 h at 37°C with 5% CO_2_. For some experiments, divalent or trivalent iron ions (250 μM) were additionally added. After incubation, the cells were fixed by adding PFA to each well at a final concentration of 4% for 15 min at room temperature and kept at 4°C until subsequent immunostaining.

### Immunostaining of H2A–H2B–DNA complex for NET visualization

Fixed cells were washed three times with PBS, blocked and permeabilized with 2% BSA PBS+0.2% Triton X-100 for 45 min at room temperature. Incubation with a mouse monoclonal anti-H2A–H2B–DNA complex (clone PL2-6, 0.5 μg/ml) in 2% BSA PBS+0.2% Triton X-100 was carried out overnight at 4°C as previously described [15]. Samples were washed with PBS and subsequently incubated with an Alexa-Fluor-488-labelled goat-anti-mouse IgG antibody for 45 min at room temperature. After washing, slides were mounted in ProlongGold® antifade with DAPI (Invitrogen) and analysed by confocal fluorescence microscopy using a Leica TCS SP5 confocal microscope with a HCX PL APO 40x 0.75–1.25 oil immersion objective. Settings were adjusted in accordance to control preparations using an isotype control antibody.

Due to donor-specific variations in spontaneous NET-release, each experiment was performed with neutrophils derived from a minimum of three independent healthy blood donors. For each preparation, a minimum of six randomly selected images were acquired per slide and used for quantification of NET-producing cells. Data are expressed as percentages of NET-forming cells in relation to the total number of cells visualized with DAPI to stain the nuclei.

### NET entrapment assay

Bacteria were grown in brain-heart infusion (BHI) medium at 37°C under agitation. Fresh overnight cultures were diluted 1:100 in BHI and then grown to mid-exponential growth phase (*D*_600_=0.7). The bacteria were washed and FITC (0.33 mg/ml, Sigma–Aldrich) labelled for 30 min in the dark. Subsequently, neutrophils were infected for 90 min at 37°C and 5% CO_2_. After incubation, non-entrapped bacteria were washed away and bacterial entrapment within the NETs was analysed by measuring the fluorescence signal at 485/538 nm compared with total amount of bacteria. The FITC signal was measured and entrapped cfu/well were calculated based on a standard curve with FITC-labelled bacteria. Percentage of entrapped bacteria was calculated compared with total amount of bacteria.

### Visualization of cell death

For microscopic examination of cell death, cells were cultured on glass-bottom culture plates and analysed for viability using the LIVE/DEAD viability/cytotoxicity kit for mammalian cells (Invitrogen) following the recommendations of the manufacturer.

### Immunostaining of PAD4 for PAD4 quantification and NET visualization

The procedure is the same as described above but additional antibodies were used. A polyclonal rabbit anti PAD4 antibody [22] was used as a primary antibody besides the respective isotype IgG rabbit (Jackson Immunoresearch) as control staining. As the additional secondary antibody Alexa Fluor 633 goat anti rabbit (Invitrogen) was used.

ImageJ software was used for quantification of the PAD4-expression. Thus, the fluorescent intensity caused by the excited secondary antibody Alexa Fluor 633 which binds to the anti PAD4 antibody was compared with the intensity of the respective isotype control.

### Blocking activity of PAD4, NADPH oxidase or elastase

Human blood-derived neutrophils (isolation as described above) were treated with chloramidine [16] in a final concentration of 200 μM to block PAD4-activity, with 10 μg/ml diphenylene iodonium (DPI) to block NADPH-oxidase-dependent formation of ROS, 40 μg/ml aprotinin or 0.1 mM elastase inhibitor *N*-(methoxysuccinyl)-Ala-Ala-Pro-Val-chloromethyl ketone (EnzChek® Elastase Assay Kit E-12056, Invitrogen) to block elastase at the same time when stimulating the cells with the iron chelators as NET inducers. The stimulation itself as well as incubation and subsequent fixation were performed as described above.

### PAD4 detection in Western blot analysis

Neutrophils have been isolated and stimulated as described above. The cells were lysed in standard lysis buffer with proteinase inhibitors and proteins were separated via SDS/10% PAGE. After electrophoresis, proteins were transferred on to a PVDF membrane for 100 min at 240 mA and blocked in 0.1% TBST+5% nonfat dry milk for 45 min. For PAD4 detection, the blot was incubated with the polyclonal rabbit anti-PAD4 antibody [22] over night at 4°C with agitation. Additionally a monoclonal mouse anti-β-actin antibody (Santa Cruz Biotechnology) was used as a loading control. The respective secondary antibody (goat anti-rabbit HRP and goat anti-mouse HRP) was added for 45 min at RT with agitation. Proteins were detected using SuperSignal West Femto Chemiluminescent Substrate reagents (Pierce, Thermo Scientific). The software ImageJ was used for signal quantification. Therefore, the PAD4 signal was normalized against the β-actin control signal.

### Statistical analysis

For statistical analysis GraphPad Prism 5.0 (Graph Pad Software) was used. Data derived from a minimum of three independent experiments were analysed. For analysis of time-dependent effect of DFO on NET-formation, two-way ANOVA, followed by a Sidak's multiple comparison to control group (no matching) was used. For concentration-dependent effect of DFO on NET-formation, non-parametric Kruskal–Wallis test followed by a Dunn's multiple comparison to control group (no matching) was applied. For all other data a student's *t* test (paired/non-paired, one-tailed) was performed. A paired *t* test was chosen when the respective data (test samples and controls) have been matched within the experimental setting (Figures 3 and 6C). For all tests *P* was defined as *P<0.05; **P<0.005; ***P<0.001, ****P<0.0001.

## RESULTS

### Iron-chelating agent DFO mediate formation of NETs

**Figure 1:**
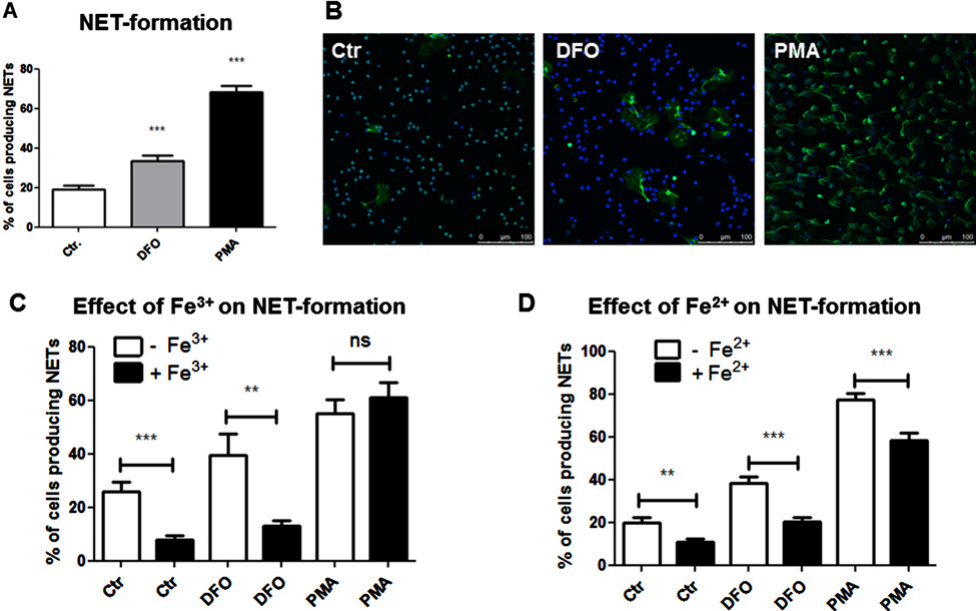
DFO induces NET formation in human neutrophils Human blood-derived neutrophils were isolated by density gradient centrifugation and treated with DFO in the presence and absence of excessive iron ions. Formation of NETs was visualized using the PL2–6 antibody against H2A–H2B–DNA complexes (green) in combination with DAPI to stain the nuclei (blue). (**A**) Increased NET formation was detected for neutrophils stimulated for 3 h with 300 μM DFO or 25 nM PMA as positive control. (**B**) Representative fluorescent micrographs of human neutrophils incubated in media only, media containing 300 μM DFO or 25 nM PMA as positive control representing the results of the column bar graph in (**A**). (**C** and **D**) Addition of divalent or trivalent iron ions (250 μM) abolishes the NET-induction effect by DFO. The graphs represent the mean ± S.E.M. of 60 images from 10 independent experiments (**A**) or 18 images derived from three independent experiments (**C** and **D**).

To examine the effect of iron chelation on NET formation, human blood-derived neutrophils were treated with the iron-chelating agents DFO (300 μM) for 3 h at 37°C and 5% CO_2_ and percentage of NET-releasing cells was quantified compared with untreated control; 25 nM PMA was used as positive control as previously shown [6]. As shown in Figure 1(A), DFO treatment of cells showed a modest but significant induction of NET formation compared with the negative control group. Representative immunofluorescent micrographs of DFO-mediated NET induction compared with its negative and positive control are shown in Figure 1(B), with NET structures visible as histone-DNA extrusions of the nuclei of the cells. Figures 1(C) and 1(D) corroborate that NET formation was related to the iron chelation activity of the drug, as addition of excess iron decreased the induction phenotype by DFO; both ferrous and ferric forms of iron (Fe^2+^ or Fe^3+^) supplementation had the same effect. However, the dose of iron ions (250 μM) appears to be insufficient to completely abolish the effect of DFO (300 μM; Figures 1C and 1D), because DFO binds to iron ion at one-to-one ratio. Notably, a decreased NET induction phenotype in PMA stimulated cells was only visible for an excess of Fe^2+^.

**Figure 2:**
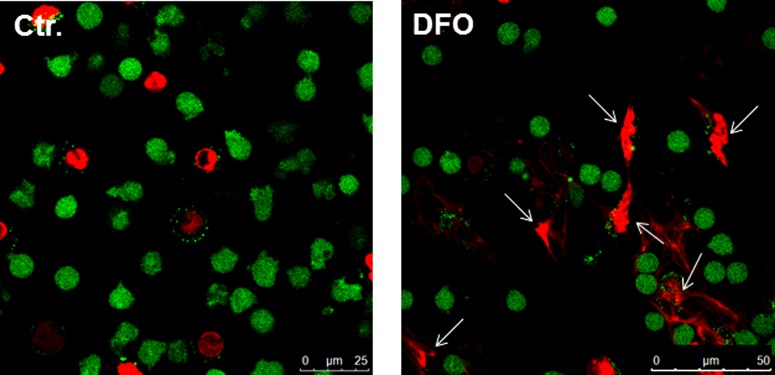
Determination of live (green) and dead (red) neutrophils in the presence of DFO For microscopic examination of cell death, cells were cultured on glass-bottom culture plates and analysed for viability using the LIVE/DEAD viability/cytotoxicity kit for mammalian cells (Invitrogen) following the recommendations of the manufacturer. Note the release of NETs by dying neutrophils (red) in the representative immunofluorescence (white arrows).

Classically, NET formation has been shown to be the result of a cell death mechanism associated with the extracellular release of nuclear DNA, called NETosis [6]. But it is important to mention that some authors have experimentally demonstrated that neutrophils can also release NETs in response to infection while remaining in a viable status [23]. Using immunofluorescence microscopy we confirmed that NET-forming cells in response to DFO are dying (Figure 2). Interestingly, DFO appears to selectively induce NETosis as compared with control, because there are only random cases of dead cells visible showing no NETosis (representative Figure 2).

**Figure 3:**
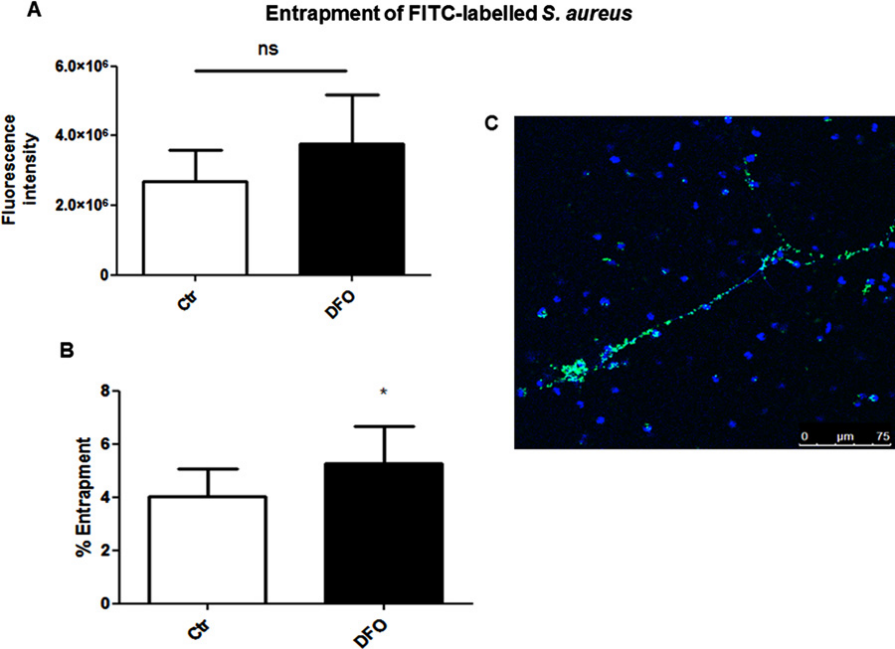
DFO induces entrapment of *S. aureus* in NETs released by human neutrophils (**A**) Human blood-derived neutrophils were isolated by density gradient centrifugation, seeded, stimulated with media only or media containing 300 μM DFO for 2 h, infected with FITC-labelled *S. aureus* (MOI=10) for 90 min and washed to remove unbound bacteria. The FITC signal was measured and entrapped cfu/well were calculated based on a standard curve with FITC-labelled bacteria. (**B**) Percentage of entrapped bacteria was calculated compared with total amount of bacteria. The data in (**A**) were used as basis for the calculation of percentage of entrapment in (**B**). Data are shown as mean ± S.E.M. of four independent experiments. (**C**) Representative fluorescent micrograph of FITC-labelled *S. aureus* (green) entrapped in DAPI stained DFO-induced human NETs (blue).

**Figure 4:**
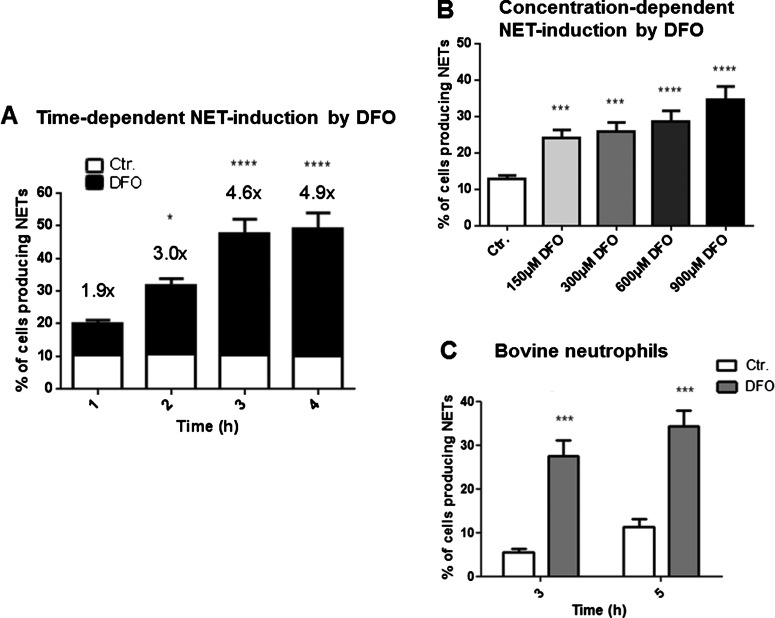
DFO-induced NET formation is time- and concentration dependent and not limited to human neutrophils Human and bovine blood-derived neutrophils were isolated by density gradient centrifugation, stimulated and the formation of NETs was visualized using the PL2–6 antibody against H2A–H2B–DNA complexes (green) in combination with DAPI to stain the nuclei (blue). (**A**) Human neutrophils were stimulated with 300 μM DFO for 1, 2, 3 and 4 h and subsequently fixed in 4% PFA. NET formation was determined in comparison with the unstimulated control. A significant increase in the amount of cells that form NETs was observed over time. The numbers on top of the bars represent the fold increase in NET-release from cells treated with DFO compared with the unstimulated control. (**B**) Different DFO concentrations (150, 300, 600, 900 μM) were tested on their ability to induce NETs in human neutrophils after an incubation period of 3 h. (**C**) NET formation in bovine neutrophils after stimulation with either media only or media containing 300 μM DFO for 3 and 5 h. The graphs represent the mean ± S.E.M. of the 24 (**A**), 30 (**B**), 12 (**C**) images derived from 4 (**A**), 5 (**B**), 2 (**C**) independent experiments.

To confirm that NETs induced by iron chelation are functional, we demonstrated that extracellular entrapment of methicillin-resistant *S. aureus* (MRSA, USA300 strain) was increased after neutrophil treatment with DFO (Figure 3). DFO-mediated NET induction was both time and concentration dependent (Figures 4A and 4B), and a similar effect was documented in bovine derived neutrophils, indicating that the DFO-mediated NET induction is not restricted to human cells (Figure 4C).

### ROS and proteases contribute to DFO-mediated NET formation

**Figure 5:**
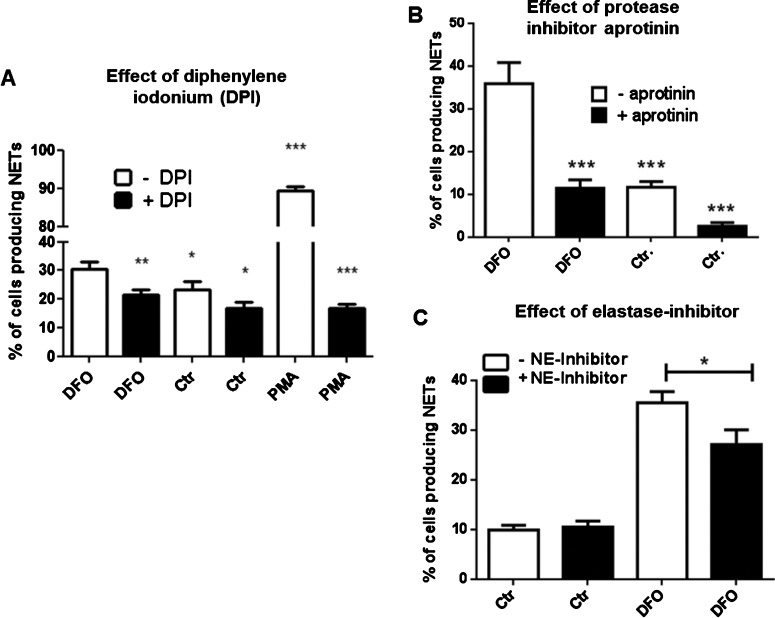
NADPH-oxidase and elastase contribute to DFO-mediated NET formation Human blood-derived neutrophils were isolated by density gradient centrifugation and NET formation was visualized using the PL2–6 antibody against H2A–H2B–DNA complexes (green) in combination with DAPI to stain the nuclei (blue). (**A**) Cells were incubated for 3 h in media only or media containing 300 μM DFO or 25 nM PMA in the presence and absence of DPI (10 μg/ml). (**B**) Cells were incubated for 3 h in media only or media containing 300 μM DFO in the presence and absence of aprotinin (40 μg/ml). (**C**) Cells were incubated for 3 h in media only or media containing 300 μM DFO in the presence and absence of 0.1 mM elastase inhibitor *N*-(methoxysuccinyl)-Ala-Ala-Pro-Val-chloromethyl ketone. All graphs represent the mean ± S.E.M. of a minimum of 18 images derived from three independent experiments.

Since NADPH-oxidase-dependent formation of ROS has been shown to contribute to NET formation [6,24], DPI was used to inhibit NADPH oxidases to test the role of NADPH oxidases in DFO-induced NET formation in human neutrophils. As shown in Figure 5(A), we found that DPI significantly blocks the formation of NETs, indicating that a NADPH-oxidase-dependent process of NET formation is induced by DFO.

Recently, the proteolytic activity of neutrophil elastase was shown to modify histones and contribute to NET formation [17]. The trypsin inhibitor and anti-fibrinolytic drug, aprotinin, has further been shown to block the activity of serine proteases such as neutrophil elastase [25]. As shown in Figure 5(B), treatment of neutrophils with the general serine protease inhibitor aprotinin as well as the more specific elastase inhibitor *N*-(methoxysuccinyl)-Ala-Ala-Pro-Val-chloromethyl ketone [26] (Figure 5C) also significantly blocked the DFO-mediated NET formation, indicating that the activity of serine proteases (e.g. elastase and others) is also partially involved in the induction of this phenotype.

### PAD4 is partially involved in DFO-induced NET formation

**Figure 6:**
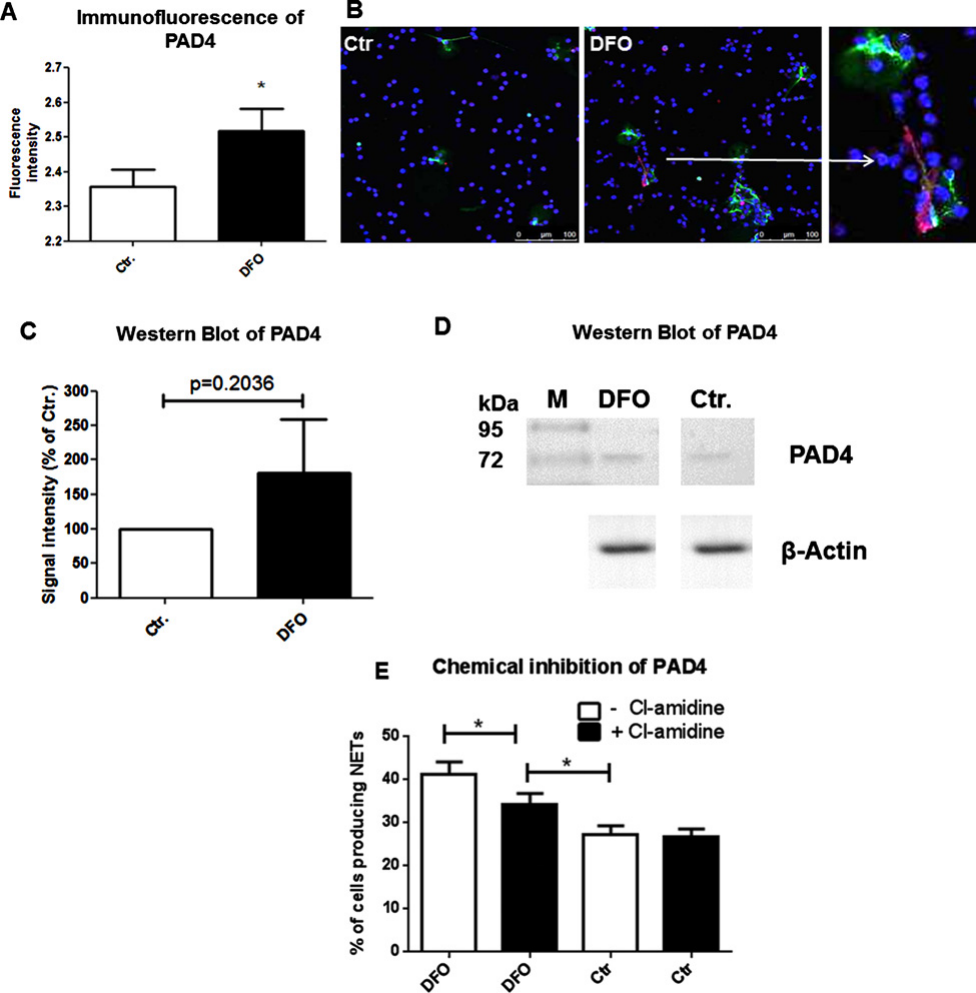
DFO increases PAD4 protein level and Cl-amidine diminishes the DFO-mediated NET-induction effect PAD4 protein level was quantified in human blood-derived neutrophils treated with 300 μM DFO in comparison with untreated neutrophils using immunofluorescence microscopy and Western blotting. (**A**) The fluorescence intensity of the PAD4 signal was measured using ImageJ. Statistical analysis was performed using 24 images of four independent experiments. (**B**) It shows representative immunofluorescent micrographs of neutrophils stained with an antibody to visualize PAD4 (red) within the NET structures (green). DAPI was used to counterstain the nuclei in blue. A higher level of PAD4 was detected in cells that release NETs and that were treated with the iron chelator DFO (**C**) compared with the untreated control (**B**). (**C**) Signal intensities of PAD4 in control and DFO stimulated cells observed in three independent Western blot experiments. Data are indicated relative to a β-actin control. (**D**) Representative Western blot of data shown in (**C**). (**E**) Neutrophils were treated with or without 200 μM Cl-amidine and stimulated with media only or media containing 300 μM DFO for 3 h. This graph represents the mean ± S.E.M. of 18 images derived from three independent experiments.

Hypercitrullination of histones by PAD4 was shown to be a key step in chromatin unpacking during NET formation induced by TNFα [16]. We quantified PAD4-expression in DFO-treated human blood-derived neutrophils in comparison with untreated neutrophils using immunofluorescence microscopy (Figure 6A). An increased level of PAD4 was present in cells treated with the iron chelator DFO. Figure 6(B) shows representative immunofluorescence micrographs of neutrophils stained with a DNA–histone-complex antibody (green) in combination with a PAD4 antibody (red) to visualize PAD4 in NET structures as well as DAPI (blue) to visualize the nucleus. Furthermore, we used semiquantitative Western blot analysis, which only showed a trend towards higher PAD4-protein content in DFO-treated cells (*P*=0.2; Figures 6C and 6D). Next, PAD4-activity was blocked by addition of the known inhibitor chloramidine (Cl-amidine) [16,27]. In the presence of Cl-amidine, a significant reduction in DFO-induced NETs was observed, though this level still exceeded that in untreated control cells (Figure 6E). These data suggest that PAD4-mediated histone modifications partially contribute to the observed DFO-mediated NET formation.

## DISCUSSION

The data presented in this paper show that the tested iron chelator DFO slightly, but significantly, induces NET formation in human blood-derived neutrophils and thereby lead to modestly enhanced entrapment of *S. aureus*. Other own studies have already shown that NETs can play a protective role against *S. aureus* infections based on their ability to entrap, immobilize as well as inhibit the growth of the bacteria [3,21]. Thus, our data are consistent with a previous publication showing that the iron chelator mimosine can boost the antimicrobial effect of neutrophils against *S. aureus* infections [19]. When cytochalasin D was used by those authors to inhibit phagocytosis, enhancement of killing by mimosine was still observed, suggesting that the bactericidal effectors induced by mimosine can function extracellularly [19]. Zinkernagel et al. [19] showed that triggering of neutrophil killing by mimosine was eliminated by treatment with DNase, which degrade NETs, thus assuming that mimosine induces NETs and thereby may trigger NET-mediated antimicrobial activity against *S. aureus*. Here we confirm that besides DFO also the iron chelator mimosine is able to induce formation of NETs (Supplementary Figure S1).

Some important key mechanisms that have been identified to be involved in NET formation are the NADPH-dependent formation of ROS [6], the elastase-mediated histone degradation [17] or the PAD4-mediated histone hypercitrullination [16]. When blocking the respective enzymes biochemically during our assay, we could significantly diminish the DFO-mediated NET formation. These data indicate that all those enzymes are involved in the process.

The DFO-mediated NET formation can also be abolished by an iron excess mediated by supplementation with Fe^2+^ or Fe^3+^. If abnormalities in NET formation are detectable in patients suffering from chronic iron deficiency anaemia or iron overload (e.g. hemochromatosis) still remains to be determined. Improper regulation of NET formation may contribute to sepsis, systemic inflammatory response syndrome, small vessel vasculitis or vascular injury associated with systemic lupus erythematosus [28–31].

Iron is an essential factor involved in the general stress response of a cell by regulating activity of key enzymes such as prolyl hydroxylases (PHD). Iron- and oxygen-dependent PHDs are the key factors responsible for the degradation of the α subunit of the hypoxia inducible factor HIF-1 during normoxia [32]. During hypoxia or following an acute inflammatory stimulus, PHD-mediated degradation of HIF-1α is reduced [33] leading to altered gene regulation in the cell [33,34]. An Fe(II) ion is located within the catalytic site of PHDs and is coordinated by one aspartate residue and two histidine residues [35]. Proline and asparagine residues of HIF-1α are hydroxylated by PHDs, which enables HIF-1α to bind to the von Hippel–Lindau tumour suppressor protein (vHL), a protein with ubiquitin ligase activity (Supplementary Figure S2). As a consequence HIF-1α is no longer able to bind to coactivators such as CREB-binding protein (CBP) and p300 and in the end gets ubiquitinated and thereby labelled for 26S proteasomal degradation [36–38]. Under hypoxic conditions or iron-limited conditions, HIF-1α is accumulating due to interruption of the degradation pathway by inhibition of the PHD-mediated hydroxylation. The heterodimeric transcription factor HIF-1 is formed and is able to interact with its coactivators which leads to binding of HIF-1 to specific binding sites, so called hypoxia responsive elements (HREs) [39–42]. HIF-1 binding regulates the transcription of target genes that encode erythropoietin, glucose transporters, glycolytic enzymes, antimicrobial factors and the angiogenic factor VEGF [40,42].

By virtue of their iron chelation, DFO and L-mimosine are known HIF-1α agonists, but in case of mimosine with less iron-chelating capacities [35,19,42–50]. HIF-1α regulates elastase and NADPH-oxidase expression on transcriptional level [41] and HRE-binding sites [51] are found in the promoter region of PAD4 (Supplementary Figure S3). Thus, HIF-1α-mediated activation of those enzymes might represent a key trigger for the NET formation induced by DFO. In line with this hypothesis, we were also able to show that the HIF-1α-protein stabilizing agents cobalt chloride, L-mimosine, AKB 4924 or dimethyloxalylglycine (DMOG) [52–56] showed modest but statistically significant increase in NET formation (Supplementary Figure S1). Recently, we have observed that the HIF-1α-agonist AKB 4924 also facilitated the formation of mast cell extracellular traps (MCETs) [57] in murine and human mast cells. Others recently reported that HIF-1α contributes to rapamycin-induced NET formation in human leukaemic HL-60 cells [58]. Thus, it may be hypothesized that stabilization of HIF-1α might facilitate formation of ETs in myeloid cells in hypoxic or iron-deficient tissue as it occurs during infection [40,59–61].

In conclusion, our study shows that iron-chelating agent DFO slightly boosts the formation of NETs in human primary blood-derived neutrophils, an effect that can be abolished by iron supplementation. Since DFO is described as well-known iron-chelating prolyl hydroxylase inhibitors, our data support the hypothesis of other recent publications with mast cells and a human leukaemic cell line (HL-60 cells) that blockage of prolyl hydroxylases may facilitate formation of NETs. Iron-chelating prolyl hydroxylase inhibitors are in advanced clinical development for anaemia therapy, and might be explored in a novel context of NET induction to support innate immune clearance of problematic pathogens. Although the formation of NETs is frequently associated with a protective effect against microbial infections, increasing evidence is given that an excessive release of NETs has been associated with detrimental consequences for the host, e.g. autoimmune diseases [18]. Thus, a fine balance between NET formation and NET degradation by the host itself seems to be essential for a final protective outcome of an infectious disease and iron could be an essential factor for a pharmacological manipulation of the formation of NETs in both directions depending on the disease status of the patient.

## Abbreviations

BHI: brain-heart infusion
DFO: desferrioxamine
DMOG: dimethyloxalylglycine
DPI: diphenylene iodonium
ET: extracellular trap
HRE: hypoxia responsive element
IL-8: interleukin-8
NET: neutrophil extracellular trap
PAD4: peptidyl-arginine-deiminase 4
PMA: phorbol myristate acetate
ROS: reactive oxygen species
